# Distribution and determinants of young child feeding practices in the East African region: demographic health survey data analysis from 2008-2011

**DOI:** 10.1186/s41043-015-0008-y

**Published:** 2015-05-01

**Authors:** Constance A Gewa, Timothy F Leslie

**Affiliations:** 1 Department of Nutrition and Food Studies, George Mason University, 4400 University Dr. MSN 1F8, Fairfax, VA 22030 USA; 2 Department of Geography and Geoinformation Science, George Mason University, 4400 University Dr. MSN 6C3, Fairfax, VA 22030 USA

**Keywords:** Infant and young child feeding, Complementary feeding, Kenya, Uganda, Tanzania, East Africa

## Abstract

We utilized the most recent Demographic Health Survey data to explore the distribution of feeding practices and examine relationships between complementary feeding and socio-demographic and health behaviour indicators in Kenya, Uganda and Tanzania. We based our analysis on complementary dietary diversity scores calculated for children 6-23 months old. Geographically, Kenya displayed clear division of children’s diet diversity scores across its regions, unlike Uganda and Tanzania. Less than 40% of the children’s meal frequencies in Uganda and Tanzania had met the minimum daily recommended levels. Only 30-40% of children in Kenya, Tanzania and Uganda had consumed diets with adequate diversity. Children’s age, breastfeeding status, mother’s education level and working status, household wealth index, prenatal care visits, receiving vitamin A supplements, using modern contraceptives and meal frequencies were significantly associated with adequate complementary food diversity in at least one of the three countries included in the current analyses. These analyses contribute to a better understanding and targeting of infant and young child feeding within the East African region.

## Background

Undernutrition accounts for 35% of deaths among children less than five years of age, with a vast majority of these losses reported in Africa and Asia [1]. The first two years of life is a critical period characterized by high rates of growth and development and accompanying high nutritional requirements [2]. Research has shown that exclusive breast-feeding adequately provides for children’s energy and nutrient needs in the first six months of life [3]. However breast-milk alone cannot meet the increased energy and nutrient requirements as children get older [3]. Thus, the World Health Organization (WHO) recommends that infants should be exclusively breastfed for the first 6 months of life, after which they are introduced to appropriate complementary foods as they continue to breastfeed [4,5]. Consumption of adequate non-breast-milk foods contribute to improved growth, health and development of young children [6-9]. The WHO recommendations call for timely introduction of nutritionally adequate, appropriate, and safe foods for all children, and further indicate that a variety of foods should be consumed to ensure children’s nutrient needs are met [10]. Furthermore, animal-source foods and vitamin A-rich fruits and vegetables should be eaten daily or as often as possible [10]. While the recommendations do not vary across countries, the application of these recommendations is more critical in low-income nations that bear the larger burden of child malnutrition and limited resources [11-13].

In this paper, we investigated infant and young child feeding (IYCF) within three countries in East Africa: Kenya, Uganda and Tanzania. These countries have an unusually strong relationship besides their shared borders. They have similar weather and geological patterns with agriculture and tourism forming the backbone of their economies and have been long-term trade partners and consult each other on matters regarding policy, trade, and marketing [14]. Despite these similarities, child malnutrition rates vary greatly across the three countries [15]. With respect to child nutrition, Uganda has experienced a substantial decline in stunting prevalence from 45% in 2000/01 to 33% in 2011, while stunting rates have remained unchanged at above 40% in Kenya and above 35% in Tanzania over a similar time-period [15]. Understanding the role of various factors in determining IYCF contributes to knowledge needed in achieving improved child nutrition in these countries. Within the past several years, all three countries have defined policies to protect, promote and support appropriate IYCF practices [16-20]. Translation of policies to actual practice is complex and is often influenced by both micro-and macro-level factors [21]. Understanding the determinants of IYCF would help contribute to knowledge needed to improve child feeding environment and child nutrition in these countries. To our knowledge, documented studies on determinants of infant and young children’s DDS are generally lacking in Uganda and Tanzania while the more recent studies that have been conducted in Kenya have focused on specific regions within the country [22,23]. There is need to update the literature on this region using recent national sample data. Our objectives were to assess the geographical distribution of complementary feeding practices, and examine the relationship between select socio-economic, demographic and health-care utilization indicators, and complementary feeding practices within Kenya, Uganda and Tanzania.

## Methods

This analysis utilized secondary data from the standard DHS data collected nationwide in Kenya in 2008/09, Uganda in 2011, and Tanzania in 2010. Within each country, representative probability samples were selected by DHS using a multistage stratified cluster sampling methodology [15]. The DHS sample sizes were calculated to account for separate key indicators [15,24]. Clusters were selected from the master frames in the first stage via the probability proportion to size method. Households were then selected from a sampling frame using a random systematic method [15,25]. Representative samples of 10000, 10086 and 10300 households were drawn for surveys in Kenya, Uganda and Tanzania respectively [15]. Informed consent was obtained from each lead respondent, within the household, prior to questionnaire administration [15]. The ICF (Inner City Fund) International’s Institutional Review Board approved the DHS protocol. Survey response rates fell between 96% and 99% across the years for all countries of interest. Data was weighted to cater for different sample proportions across regions.

**Figure 1:**
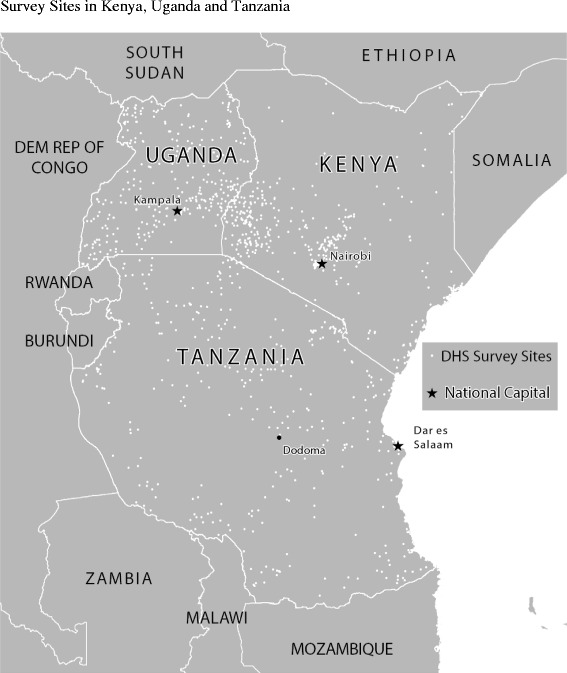
Survey Sites in Kenya, Uganda and Tanzania.

Figure 1 portrays the mapped distribution of DHS collection points. Uganda’s sampling is the densest, avoiding only Lake Victoria in the southeast. Tanzania has several large game reserves with no sampled populations. Kenya has clear regional selections. In Kenya, almost all DHS observations were taken from the central and southwestern parts of the country. Included on the figures are the countries’ capital cities; for Tanzania, we referenced both the commercial and administrative capitals, Dar es Salaam and Dodoma.

### Geographic distribution

We used cartographic analysis to investigate the spatial trends of diet variety across East Africa. Similar to previous research, we interpolated a surface with the most recent data available across the three countries using a Kriging method, a common interpolation method used to provide a regional understanding of dataset-wide trends [26,27]. We calculated the percent of consumption of individual food groups and the mean DDS for each of the 1162 “cluster” location, and then used these scores to estimate diet variety scores for every location using nearby DHS survey sites. The number of individual observations per cluster ranged from 1 to 17, with an average of 3.9. Although the exact locations of the survey cites were somewhat displaced to preserve respondent anonymity, the final surface incorporated an error term that mitigates the bias introduced by DHS [28].

### Complementary feeding practices

Information on complementary foods consumed by children in the previous 24 hours was available for children between the ages of 0 and 24 months. Caregivers, mostly mothers, were asked to indicate if the target child had consumed selected complementary food within the last 24 hours.

### Dietary diversity

The WHO recommends the use of seven food groups namely grains/roots/tubers; legumes and nuts; dairy products; flesh foods (meats/fish/poultry); eggs; vitamin A-rich fruits and vegetables (VAFV); and other fruits and vegetables (OFV) in defining children’s dietary diversity score [29]. However, because the flesh foods and eggs had been combined as a single entry in the Kenyan DHS survey, a decision was made to use the combined meats/fish/poultry/eggs (MFPEs) food group for children in all three countries. Thus the children’s complementary food dietary diversity score (DDS) definition was based on six food groups: (i) grains/roots/tubers, (ii) VAFVs, (iii) OFVs, (iv) legumes and nuts, (v) dairy, including formula and (vi) MFPEs. The DDS was constructed by assigning one point to each of the defined food groups, for a maximum of six points. Because the MFPs and eggs were collected as separate variables in the Uganda and Tanzania surveys, a decision was made to assign one point to the MFPEs food group if a child had consumed either MFPs or eggs on the day of reference.

The DDS is a simple but valid dietary assessment tool that has been shown to be a good indicator of micronutrient intake among young children [7,30-32]. Consumption of foods from at least 4 food groups has been associated a high likelihood of a child consuming at least one animal-source food and at least one fruit or vegetable, in addition to a staple food and, has been used to classify diets of children from developing countries [29,33] Drawing from this literature, we defined adequate complementary food dietary diversity as consumption of food from at least four different food groups (DDS ≥ 4).

### Information collected

#### Child factors

Information was collected on children’s ages, birth order, sex, breast-feeding status and morbidity experience. Children’s ages were categorized into 9-11, 12-17 and 18-24 months to allow for comparison with previous studies [29,32]. Mothers were asked if the children were breast-feeding at the time of the survey. For morbidity experience, respondents were asked to recall if the child had experienced any diarrhea or cough episode in the previous 24 hours or within the two weeks preceding the interview.

#### Socio-economic and demographic factors

The sex of the household head was identified in the survey. Information was collected on mothers’ age, marital status (married, living with partner, or not married at time of survey) and highest level of education (no school/preschool, primary school, secondary school and above) attained by the mothers. Household socio-economic status was determined from the DHS wealth index. The DHS household wealth index is a standardized asset-based score that is divided into quintiles from one (lowest) to five (highest) [34]. Additional household variables included household residence (urban/rural) and household size (total number of people who usually lived in the household).

#### Health utilization/behaviour factors

Health utilization/behaviour factors included number of prenatal care visits (PNC) and child’s immunization status, receipt of vitamin A supplements and meal frequency. Mothers were asked to indicate the number of PNC visits made to a health care provider while pregnant with the respective child. The WHO recommends a minimum of four pre-natal care visits among pregnant women and a cut-off of at least four visits was used to identify mothers with adequate PNC visits [35]. Information on immunization coverage was collected from children’s health records and respondents’ reports. Specific vaccines included Bacillus Calmette-Guérin (BCG), birth polio, polio I-III, diphtheria-pertussis-tetanus (DPT) I-III, and measles vaccine. An indicator of completion of age-specific immunization was created based on the recommended immunization schedule for infants in developing countries [36]. Information on Hepatitis B vaccination was not available. Information on receipt of vitamin A supplements was collected from children’s health records. The WHO recommends that, in settings where vitamin A deficiency is a public health problem, children 6-11 months should receive one dose of 100,000 IU (30 mg RE) vitamin A, while children 12-59 months should receive 100,000 IU (30 mg RE) vitamin A, every 4-6 months [37].

Mothers were asked to indicate the number of times, in the previous day and night, a child had received anything to eat, aside from breast-milk, including meals and snacks. The WHO’s minimum meal frequency definitions were applied [29]. Frequencies of consumption of solid, semi-solid and soft foods (including milk feeds for non-breastfed children) were calculated. For children who were still breast-feeding at the time of the surveys, minimum frequency was defined as 2 times for infants 6–8 months and 3 times for children 9–24 months [29]. For children who had stopped breast-feeding by the time of the surveys, minimum frequency was defined as 4 times for children 6–24 months [29].

### Statistical analysis

A total of 5338, 2109 and 6957 children, 0-60 months old, were included in the Kenya, Uganda and Tanzania surveys respectively. Our analysis focused on children ages 6-23 months (1700, 707, 2276 children in Kenya, Uganda, Tanzania respectively). A decision was made to include only the youngest child within this age-group from each household. This resulted in 1668 children in Kenya; 701 children in Uganda and 2247 children in Tanzania with dietary diversity data. SAS Version 9.2 (SAS Institute, Cary, NC, USA) was used for data analysis. Survey analysis procedures were appropriate for the DHS complex study design and utilized to help estimate sampling errors. SAS procedures surveyfreq, surveymeans, surveyreg and surveylogistic were used to estimate means, percentages, and odds ratios. Logistic regression analysis was used to assess the odds of achieving adequate DDS. Bivariate logistic regression was used to assess the association between each independent variable (Table 1) and our outcome variable, adequate DDS (DDS ≥ 4). Thereafter, only independent variables that showed a significant association with adequate DDS, in the bivariate analysis, were simultaneously entered into a multivariate logistic regression model. Nonresponse across variables reduced the effective sample size in the multivariate regression to 1588 in Kenya, 674 in Uganda and 2185 in Tanzania (4.80, 3.85 and 2.76 percent loss in sample size, respectively).

**Table 1:** **Health and socio-economic characteristics among children 6-24 months** ^**1**^

	**Kenya**		**Uganda**		**Tanzania**	
	**Estimate**	**SE**	**Estimate**	**SE**	**Estimate**	**SE**
Children’s age, mo (mean)	14.19	0.20	14.66	0.23	14.29	0.13
Age category, mo (%)						
6 - 8 mo	19.55	1.25	17.48	1.61	16.73	0.97
9 - 11 mo	16.45	1.29	16.12	1.57	17.63	1.09
12 - 17 mo	31.94	1.56	31.36	1.87	33.87	1.24
18 - 24 mo	32.07	1.66	35.04	2.09	31.77	1.09
Birth order (mean)	3.47	0.09	4.32	0.12	3.70	0.06
Child’s sex: male (%)	51.39	1.59	47.48	2.14	48.91	1.25
Breast-feeding (%)	81.68	1.31	76.68	1.83	84.24	1.00
Diarrhea (%)	29.00	1.69	39.27	2.08	23.31	1.26
Cough (%)	32.01	2.04	54.10	2.27	27.59	1.33
Household head sex: male (%)	72.97	1.67	76.99	1.99	83.02	0.99
Mother married (%)	83.92	1.46	87.09	1.53	84.66	0.97
Mother’s education (%)						
Preschool/none	11.99	1.67	11.23	1.25	25.16	1.56
Primary school	63.66	2.06	66.89	2.05	67.14	1.50
At least secondary school	24.34	1.83	21.87	1.85	7.70	0.79
Working mother (%)	57.11	1.99	73.93	2.30	86.83	0.90
Wealth Index (%)						
First quintile	22.87	1.88	22.49	1.99	22.19	1.32
Second quintile	20.76	1.66	21.86	1.87	24.00	1.22
Third quintile	18.20	1.65	20.48	1.77	20.67	1.17
Fourth quintile	18.38	1.94	18.91	1.76	18.05	1.46
Fifth quintile	19.78	2.72	16.26	1.55	15.09	1.33
Mother’s age, years (mean)	27.18	0.22	28.05	0.30	27.97	0.18
Household size (mean)	5.95	0.14	6.15	0.13	6.96	0.18
Urban residence (%)	20.41	2.11	12.38	1.54	20.37	1.40
Prenatal care visits (mean)	3.58	0.08	3.59	0.08	6.97	0.18
At least 4 PNC visits (%)	47.11	2.00	48.62	2.32	42.29	1.36
Immunization (%)	49.49	2.04	32.55	2.31	43.46	1.62
Received vitamin A (%)	73.89	1.74	63.19	2.19	66.32	1.46
Used modern contraceptives (%)	36.19	1.71	27.22	2.01	28.39	1.29
Minimum meal frequency (%)	67.31	2.13	39.59	2.26	34.83	1.41

SE: standard error; mo: month; PNC: prenatal care.

^1^n = 1668 in Kenya, 701 in Uganda and 2247 in Tanzania.

## Results

Table 1 gives a summary of the children’s social-economic, health, and health care utilization characteristics for each of the three countries. Children’s mean ages were below 15 months, and the mean birth order was at about four across all three countries. Approximately 50% of children were males. Approximately 80% of the children were still breast-feeding at the time of the respective surveys. Highest diarrhea and cough prevalence were reported in Uganda. Mean mother’s age was at about 28 years for all three countries. A majority of mothers had primary school level education and were working. A majority of the households were headed by males and were located in rural areas. The mean household size ranged from about six to seven household members across all countries. Mothers had made an average of three PNCs. Use of modern contraceptives was below 40% in each country. Up-to-date immunization coverage was below 50% in each country. Over 60% of children had received vitamin A supplements.

### Intake prevalence and geographic distribution

**Figure 2:**
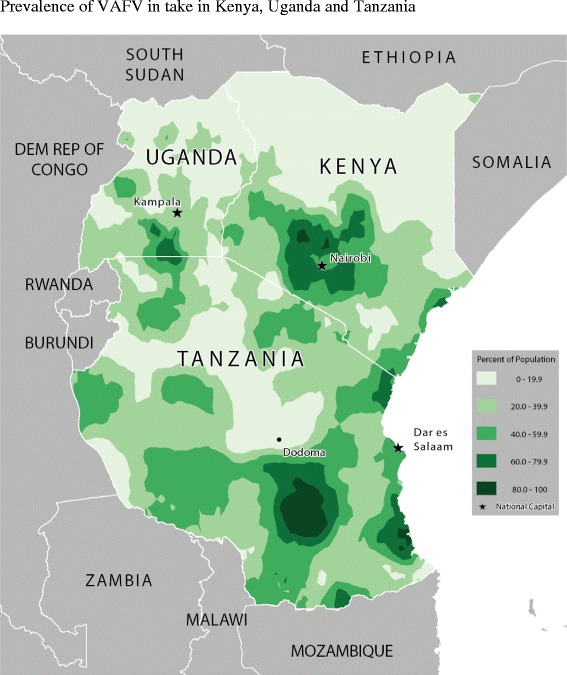
Prevalence of VAFV intake in Kenya, Uganda and Tanzania.

**Figure 3:**
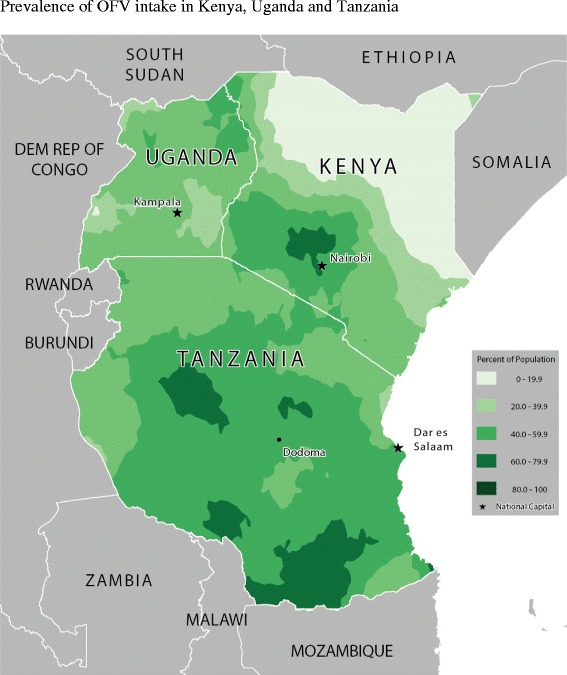
Prevalence of OFV intake in Kenya, Uganda and Tanzania.

**Figure 4:**
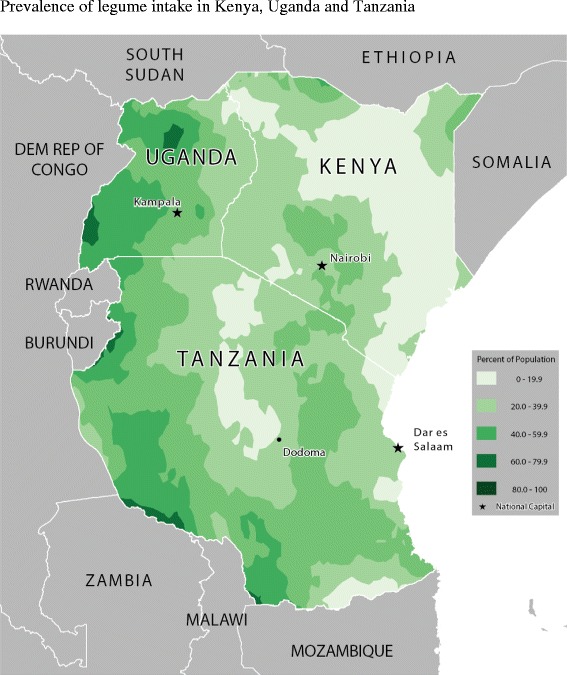
Prevalence of legume intake in Kenya, Uganda and Tanzania.

**Figure 5:**
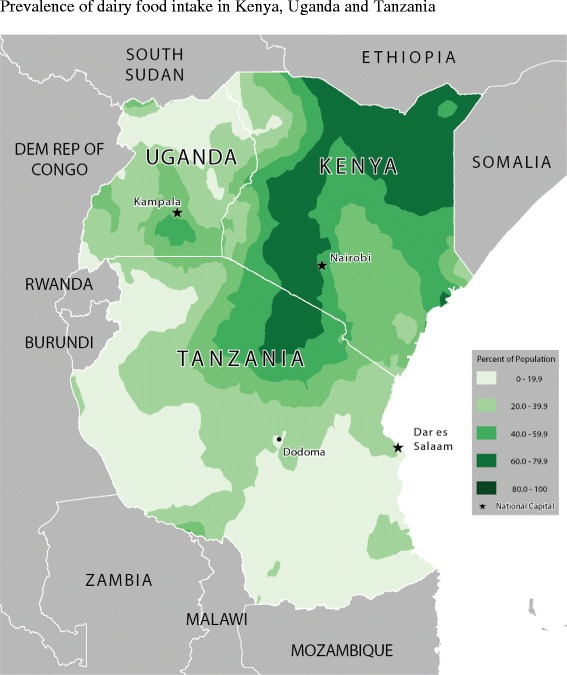
Prevalence of dairy food intake in Kenya, Uganda and Tanzania.

**Figure 6:**
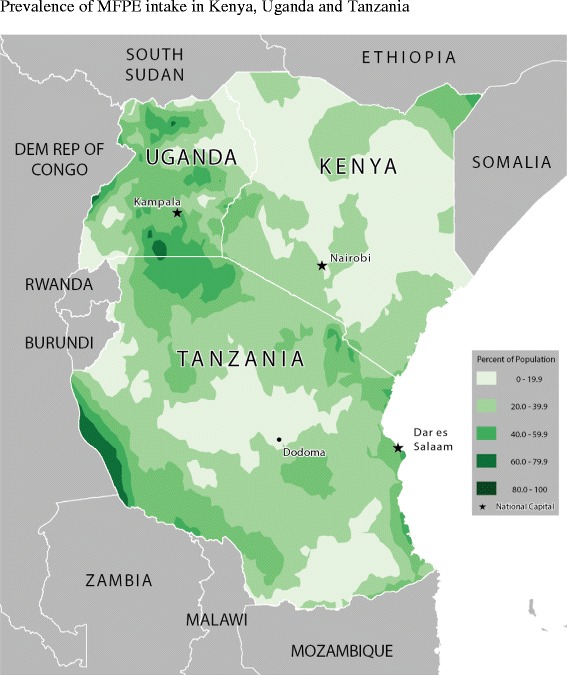
Prevalence of MFPE intake in Kenya, Uganda and Tanzania.

**Figure 7:**
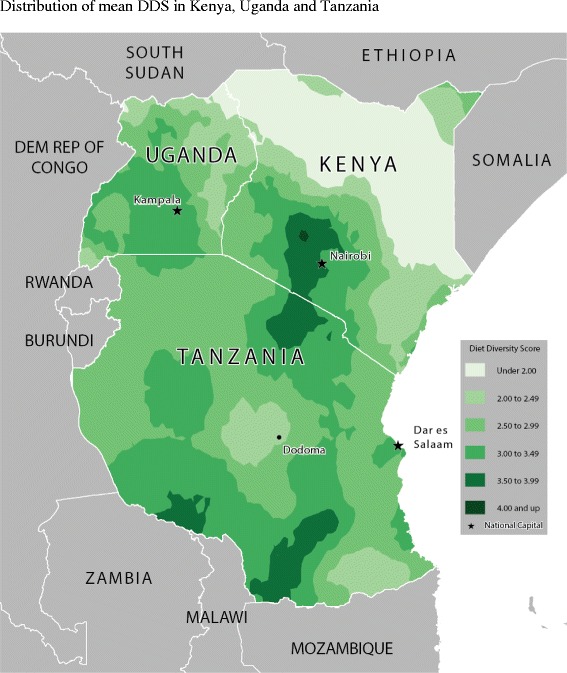
Distribution of mean DDS in Kenya, Uganda and Tanzania.

Table 2 shows the food consumption rate across age-groups and for all children. Figures 2, 3, 4, 5, 6 and 7 provide geographic representations of food group consumption rates across Kenya, Uganda and Tanzania. The percentage of children with adequate meal frequencies was higher in Kenya than in Uganda and Tanzania. The percent of children with minimum meal frequency decreased with age with the largest drop noted between the 6-8 month and 9-11 month age groups.

**Table 2:** **Meal frequency, food group consumption and DDS across children’s ages**

**Intake indicator**	**Country**	**Age of child (months)**				
		**6 -8**	**9 – 11**	**12 – 17**	**18 – 23**	**6 - 23**
Minimum meal frequency (%)	Kenya	74.86	67.74	65.39	64.15	67.31
	Uganda^*^	55.77	33.87	42.91	30.43	39.59
	Tanzania^*^	63.33	25.40	31.46	28.38	34.83
Cereals and tubers (%)	Kenya^*^	55.26	75.83	76.69	79.93	73.40
	Uganda^*^	65.04	88.56	89.75	88.45	84.78
	Tanzania^*^	83.29	92.82	93.06	94.47	91.84
VAFVs (%)	Kenya^*^	31.64	50.03	41.46	47.48	42.88
	Uganda	13.48	18.47	18.02	25.71	19.99
	Tanzania^*^	22.59	32.33	33.04	37.38	32.55
OFVs (%)	Kenya^*^	46.40	62.05	73.05	75.57	66.83
	Uganda^*^	28.59	51.32	56.73	51.33	49.05
	Tanzania^*^	44.08	62.18	67.84	65.90	62.25
Dairy (%)	Kenya	58.99	66.93	58.18	52.50	57.96
	Uganda	26.74	33.76	24.93	29.51	28.27
	Tanzania	25.99	30.55	31.40	31.36	30.34
Legumes (%)	Kenya^*^	15.19	28.52	29.26	33.62	27.79
	Uganda^*^	40.14	65.43	57.54	58.04	55.95
	Tanzania^*^	28.17	36.76	41.83	42.83	38.97
MFPEs (%)	Kenya^*^	13.39	18.65	29.47	34.58	26.19
	Uganda^*^	22.84	40.05	32.80	39.71	34.65
	Tanzania^*^	22.59	33.59	40.79	44.15	37.54
DDS < 1 (%)	Kenya^*^	14.66	4.76	5.26	7.62	7.77
	Uganda^*^	15.03	1.51	4.69	7.39	6.93
	Tanzania^*^	11.15	1.93	2.39	3.11	4.01
DDS ≥ 4 (%)	Kenya^*^	22.52	39.22	39.50	48.63	39.06
	Uganda^*^	15.45	32.09	28.73	36.00	29.50
	Tanzania^*^	18.02	30.81	34.84	38.80	32.58

^*^Statistically significant differences between age groups.

In Kenya, cereals-tubers were commonly consumed while consumption of legumes and MFPEs was lacking (Figures 4 and 6). High consumption rate (>60% of population) of VAFVs, OFVs and dairy foods was localized only to the central highlands of Kenya (Figure 2, 3 and 5). The DDS scores in Kenya showed a clear geographic partition with the highest scores observed around Nairobi and central regions (Figure 7). Score values decreased as one moved away from the central regions. An exception to this was found at the northeastern border tip which reported a mean DDS score within the 3-3.49 range (Figure 7).

With the exception of VAFVs and dairy foods (Figures 2 and 4), Uganda seemed to have fared better with a more balanced and spread-out intake of OFVs and legumes and to a certain extent MFPEs (Figures 3, 5 and 6). Uganda had fairly consistent DDS across the country with the highest DDS (≥3.50) noted around Rakai district (Figure 7).

With the exception of cereals-tubers, less than 40% of children in Tanzania had consumed foods from the remaining five food groups in the last 24 hours (Table 2). Consumption of dairy foods, VAFVs, legumes/nuts, and MFPEs in Tanzania was localized to a few specific locations within the country (Figures 2, 3, 4, 5 and 6). However, consumption of OFVs was more balanced across the country. Tanzania had fairly consistent DDS, with a pocket of low values in the central parts. The highest DDS values were noted along Tanzania’s borders with Kenya, Zambia, and Mozambique (Figure 7).

Mean DDS were below three across all countries: 2.95 ± 0.07 in Kenya, 2.73 ± 0.06 in Uganda, and 2.93 ± 0.04 in Tanzania. Kenya reported the highest percentage of children who had adequate DDS (Table 2). Tanzania reported the lowest percentage of children who had not consumed any foods from the six food groups. Food group consumption rates were significantly different across children’s age groups. With the exception of dairies, there was a distinct bump in consumption rate from the youngest children (6–8 months old) to those within the 9 –11 month age-group, after which consumption rates remained relatively stable.

### Prediction complementary food diversity

#### Child factors

Being an older child (9-11, 12-17 & 18-24 months) was associated with significantly higher odds of achieving adequate DDS compared to those within the 6-8 month age-group across all three countries: 125%-299% higher for the 9-11 month age-group, 164%-174% higher for the 12-17 month age-group, and 219%-300% higher for the 18-24 month age-group (Table 3). In Uganda, children who were breast-feeding at the time of the survey were associated with 63% reduction in odds of achieving adequate DDS compared to those who were not breast-feeding.

**Table 3:** **Predictors of adequate diet diversity (DDS ≥ 4) among children 6-23 months** ^**1**^

	**Kenya**		**Uganda**		**Tanzania**		**All countries**	
	**aOR**	**95% CI**	**aOR**	**95% CI**	**aOR**	**95% CI**	**aOR**	**95% CI**
Age category, (ref., 6 - 8 mo)								
9 - 11 mo	2.57^*^	1.47, 4.49	3.99^*^	1.81, 8.77	2.25^*^	1.40, 3.62	2.61^*^	1.87, 3.65
12 - 17 mo	2.65^*^	1.67, 4.19	2.74^*^	1.31, 5.73	2.64^*^	1.75, 3.98	2.75^*^	2.07, 3.63
18 - 24 mo	4.00^*^	2.24, 7.15	3.45^*^	1.63, 7.30	3.19^*^	2.06, 4.94	3.61^*^	2.63, 4.96
Birth order	0.95	0.87, 1.02	_	_	0.99	0.94, 1.04	0.99	0.95, 1.02
Breast-feeding, (ref., no)	0.95	0.66, 1.38	0.38^*^	0.22, 0.65	0.92	0.66, 1.38	0.85	0.67, 1.07
Cough, (ref., no)	_	_	_	_	1.26	0.98, 1.62	_	_
Education (ref., preschool/none)								
Primary school	1.59	0.84, 3.00	1.59	0.76, 3.31	1.08	0.77, 1.52	1.22	0.92, 1.62
At least secondary school	1.81	0.90, 3.66	2.10	0.87, 5.08	1.33	0.77, 2.30	1.54^*^	1.10, 2.18
Working mother (ref., no)	1.47^*^	1.03, 2.12	_	_	_	_	_	_
Wealth Index (ref., first quintile)								
Second quintile	1.72^*^	1.07, 2.75	0.64	0.32, 1.25	1.34	0.94, 1.91	1.37^*^	1.06, 1.76
Third quintile	1.64	0.98, 2.75	1.13	0.62, 2.05	2.02^*^	1.40, 2.91	1.81^*^	1.38, 2.37
Fourth quintile	2.43^*^	1.46, 4.01	1.44	0.72, 2.89	2.47^*^	1.61, 3.78	2.38^*^	1.78, 3.18
Fifth quintile	1.89^*^	1.05, 3.42	3.05^*^	1.37, 6.83	4.24^*^	2.58, 6.95	3.27^*^	2.28, 4.67
Household size (mean)^†^	0.98	0.92, 1.04	_	_	_	_	0.99	0.95, 1.03
Rural residence (ref., urban)	0.57	0.31, 1.04	1.04	0.51, 2.11	1.10	0.77, 1.56	0.88	0.65, 1.19
At least 4 PNC visits (ref., no)	2.05^*^	1.48, 2.85	_	_	1.06	0.85, 1.31	1.25^*^	1.05, 1.50
Immunized (ref., no)	0.96	0.69, 1.32	1.21	0.79, 1.85	0.98	0.78, 1.23	1.01	0.85, 1.20
Received vitamin A (ref., no)	_	_	_	_	1.43^*^	1.11, 1.84	1.12	0.92, 1.35
Modern contraceptives (ref., no)	1.31	0.92, 1.86	_	_	1.40^*^	1.10, 1.79	1.35^*^	1.12, 1.62
Min. feeding frequency (ref., no)	2.54^*^	1.80, 3.58	3.13^*^	2.06, 4.77	1.51^*^	1.21, 1.89	1.98^*^	1.67, 2.34

aOR: adjusted odds ratio; SE: standard error; mo: month; min: minimum; PNC: prenatal care.

^*^aOR estimates were statistically significant (p < 0.05), ^†^OR values computed for one additional household member.

#### Socio-economic and demographic factors

Mothers’ working status was shown to be a significant factor only in Kenya, where children whose mothers were working at the time of survey were associated with 47% increase in odds of achieving adequate DDS compared to those with non-working mothers. Children living in households within higher wealth index quintiles were associated with significantly higher odds of achieving adequate DDS compared to those living in households within the first wealth index quintile. However, the relationship was not uniform across all countries. In Kenya, significance was shown among households in the second, fourth and fifth wealth index quintiles. In Uganda, the relationship was noted among households in the fifth quintile. In Tanzania, the relationship was noted among households in third, fourth and fifth quintiles (Table 3).

#### Health utilization/behaviour factors

In Kenya, children whose mothers had made at least four PNCs were associated with 105% increase in odds of achieving adequate DDS compared to children whose mothers did not. In Tanzania, children who had received vitamin A supplements were associated with 43% increase in odds of achieving DDS compared to those had not. In Tanzania, children whose mothers utilized modern contraceptive methods were associated with 40% increase in odds of achieving adequate DDS compared to children whose mothers did not. Children who had consumed at least the defined minimum number of meals per day were associated with 153%, 213% and 51% increase in odds of achieving adequate DDS in Kenya, Uganda and Tanzania respectively (Table 3).

In a combined analysis that included all three countries, being an older child, mother’s post-primary education, higher household wealth, at least four prenatal care visits, use of modern contraceptives, and daily consumptions at or above the minimum meal frequency adequate meal frequencies were each associated with significantly higher odds of achieving adequate DDS among young children in the East African region (Table 3).

## Discussion

On average, young children’s diets in Kenya, Uganda and Tanzania consisted of foods from two food groups. Cereals and tubers have been shown to be the most common weaning food across sub-Sahara Africa [38-40]. The reported DDS means and percentages were considerably low when compared to consumption rates outside sub-Sahara Africa [7,41]. We would like to note that although the overall diet diversity scores reported in the current analysis are similar to those reported among populations in West, Central and South African regions, diet composition is expected to differ across regions [38-40]. For example, fish has been shown to be a common part of the diet in countries along the West African coastline, making MFPEs a major contributor to young children’s diet in that region [39,40].

Despite having lower mean DDS scores than Kenya, Uganda experienced a more balanced DDS distribution compared to Kenya. Over the years, Uganda has experienced an overall increase in the production of some of its major agricultural products including maize, rice and sorghum, beans, groundnuts, sesame, milk and beef [42]. However, the increased milk production did not translate to increased prevalence of consumption for this group of children. Overall, VAFVs were the least commonly consumed foods in Uganda, despite the previous promotion of orange-fleshed sweet potato as an affordable source of vitamin A in Uganda [43]. The low VAFV consumption levels may be due to the reported decline in sweet potato production in Uganda [42].

Dietary diversity score geographic distribution in Kenya seems to match the agricultural production patterns in the country; highest around the central highlands and lowest in the northern and north-eastern part of the country. An assessment of agricultural production estimates showed that the amount of maize, wheat and bean produced declined between 2003-2008 while production of red meats increased over the same period [44]. However, a large proportion of the meats consumed in Kenyan homes come from commercial sources, which tend to be quite expensive and inaccessible to those who may need them the most [34]. Among the pastoralists in the arid and semi-arid parts of Kenya, cattle are considered status symbols that help support wealth generation and maintain one’s social status and are not regularly slaughtered for home consumption. This may help explain the high consumption of dairy foods coupled with a general lack of MFPE consumption in that region.

The geographic dietary diversity score distribution in Tanzania also appears to match agricultural production patterns in the country. A recent report on food crop production in Tanzania showed that starchy foods like cassava, bananas, potatoes and maize were more common and contributed to 82% of the food crops produced over a 10 year period (1998-2008) [45]. According to the report, legumes contributed to only 5% of food crop production, 40 to 60% of the fruits and vegetables produced was lost at post-harvest and meat production from smaller animals made up less than half of the beef produced in the country [45].

Our analysis showed that consumption of foods from multiple food groups increased with age. Previous studies have documented a similar increase across age [39,40,46]. Between 70-97% of the recommended iron, zinc, phosphorous, magnesium, sodium and calcium intakes among 9-11 month old children need to be supplied by complementary foods [10]. Increasing dietary diversity is associated with a higher likelihood of meeting children’s recommended nutrient intake levels [32]. The negative relationship between breast-feeding and adequate DDS, amongst Ugandan children, has been reported in other countries and has notable implications for IYCF promotion efforts [47]. Although a majority of children within the 6-17 month age group were still breast-feeding at the time of survey, it is possible that their mothers were not aware of the importance of adequate dietary diversity in meeting young children’s nutritional needs. Although breast-milk can make a major contribution to the total nutrient intake of children within 6-24 months of age, breast-milk may not be an adequate source of micronutrients such as iron, zinc and vitamin A, as children become older, and especially in the presence of maternal deficiency [48,49].

Indicators of socio-economic status (household wealth, maternal education and a working mother) were shown to have a positive relationship with adequate DDS. Higher economic status is associated with improved access to informational, financial, and material resources. Dose-response relationships between household wealth and young children’s DDS, similar to that noted in Tanzania, has been reported in other countries [41,50]. Pre-natal care, immunization, and provision of vitamin A supplements and modern contraceptives are often offered as part of preventive care management, and caregivers have to make a conscious and informed effort to utilize them. The positive relationship between PNC visits and adequate DDS is similar to findings from India [47]. Our analysis controlled for household wealth, thus suggesting that the relationship between the use of these services and DDS goes beyond the issue of affordability. Mothers who utilized these services may be more motivated to take better care of their children. It is also likely that mothers who used these services were exposed to nutrition and health education messages. Previous research has shown that complementary feeding education among mothers was associated with higher intakes of cereals, legumes, milk, vegetables and fruits and better growth [9,51,52]. The positive relationship between meal frequencies and adequate DDS reinforces the WHO’s recommendation on complementary feeding [29]. The WHO’s meal frequency recommendations were based on children’s energy needs and gastric capacity which limits the food volume in one meal [29]. Thus, complementary food meal frequency should be addressed in nutrition education messages.

## Conclusions

Though limited by its use of one day of dietary data, our analysis has identified specific characteristics and factors that would be useful in targeting and promoting IYCN in the East African region. Inadequate complementary food diversity in all three countries, low meal frequency in Uganda and Tanzania, and to some extent delayed introduction of complementary feeding in Kenya and Uganda are of concern. We hope that results from this analysis will encourage cross-sector and cross-country collaboration to help identify specific policies and practices associated with the improving complementary food quality in all parts of the region. Adoption of nutrition-friendly health, agricultural and marketing policies that help break barriers, support increased availability and access to nutrient dense foods and expand nutrition education opportunities would help meet set IYCN goals.

## Abbreviations

BCG: Bacillus Calmette-Guérin
DDS: Complementary food diversity score
DHS: Demographic Health Survey
DPT: Diphtheria-pertussis-tetanus
FMOH: Federal Ministry of Health
IYCF: Infant and young child feeding
IYCN: Infant and young child nutrition
MFP: Meats/fish and poultry
MFPEs: Meats/fish/poultry/eggs
OFV: Other fruits and vegetables
PNC: Prenatal care
VAFV: Vitamin A-rich fruits and vegetables
WHO: World Health Organization
